# Development of a Community-Based Participatory Colorectal Cancer Screening Intervention to Address Disparities, Arkansas, 2008-2009

**Published:** 2011-02-15

**Authors:** Karen Yeary, Eric Flowers, Gemessia Ford, Desiree Burroughs, Chara Stewart, Paulette Mehta, Paul Greene, Ronda Henry-Tillman, Jackie Burton, Delores Woods

**Affiliations:** University of Arkansas for Medical Sciences, Department of Health Behavior and Health Education; University of Arkansas for Medical Sciences, Little Rock, Arkansas; University of Arkansas for Medical Sciences, Little Rock, Arkansas; University of Arkansas for Medical Sciences, Little Rock, Arkansas; University of Arkansas for Medical Sciences, Little Rock, Arkansas; University of Arkansas for Medical Sciences, Little Rock, Arkansas; University of Arkansas for Medical Sciences, Little Rock, Arkansas; University of Arkansas for Medical Sciences, Little Rock, Arkansas; Mississippi County Arkansas Economic Opportunity Commission, Inc, Blytheville, Arkansas; East Arkansas Enterprise Community, St. Francis, Arkansas

## Abstract

**Background:**

The death rate from colorectal cancer is high and affects poor and medically underserved populations disproportionately. In the United States, health disparities are particularly acute in the Lower Mississippi River Delta region. Because many in the region have limited access to basic health care resources, they are not screened for cancer, even though screening is one of the most effective strategies to prevent colorectal cancer. Community-based participatory research is a promising approach to prevent colorectal cancer in this population.

**Community Context:**

The Empowering Communities for Life program was implemented in 2 underserved counties in the Arkansas Lower Mississippi River Delta. The program arose from a 9-year partnership between the University of Arkansas for Medical Sciences and 9 cancer councils across Arkansas.

**Methods:**

Empowering Communities for Life is a community-based participatory intervention designed to increase colorectal cancer screening in rural, underserved communities through fecal occult blood testing. Community and academic partners collaborated to develop research infrastructure, intervention materials and methods, and the assessment instrument.

**Outcome:**

Project outcomes were strengthened community-academic partnerships, certification of community partners in conducting human subjects research, development of a randomized controlled design to test the intervention's efficacy, an interactive PowerPoint presentation, an informational pamphlet, the certification of 6 lay health advisors and 22 role models to provide the intervention, and an assessment tool using an audience response system.

**Interpretation:**

Lessons learned in working collaboratively with diverse groups include the importance of meeting face to face and listening.

## Background

The death rate from colorectal cancer in the United States is high (16.7/100,000) (1) and affects medically underserved populations disproportionately (2,3). Health disparities are particularly severe in the Lower Mississippi River Delta region. The region is predominately rural and has limited numbers of health care providers and facilities, low rates of health insurance coverage, low levels of educational attainment, and high rates of poverty (4,5). Because of this limited access to basic health care resources, disease management is given priority over preventive health care (4). Thus, many in the region are not screened for cancer, even though screening is one of the most effective strategies for preventing colorectal cancer (6).

By focusing on collaboration with communities disproportionately affected by disease to improve health, community-based participatory research (CBPR) is a promising approach to prevent colorectal cancer in underserved populations (7). Several CBPR studies have successfully increased screening for breast and cervical cancer (7); however, few have targeted colorectal cancer in underserved populations (7,8). The few colorectal cancer screening interventions primarily have focused on client reminders (9), which exclude people who are unable to access the health care system.

Empowering Communities for Life (ECL) uses a CBPR approach to increase colorectal cancer screening rates among rural, underserved populations in 2 Lower Mississippi River Delta counties by increasing the use of fecal occult blood testing (FOBT), a low-cost way to screen for colorectal cancer. The goal of the CBPR process used in ECL was to build infrastructure to conduct translational research, design materials and methods salient to the community, recruit and train lay health advisors and role models, and develop an assessment instrument. In this article, we describe the development of the CBPR partnership, development of ECL, and lessons learned by using a CBPR approach.

## Community Context

The community context for ECL is 2 Arkansas counties in the Lower Mississippi River Delta region, Mississippi and St. Francis. Both counties are designated as medically underserved and health professional shortage areas, and access to health care resources is further complicated by the counties' and state's decentralized and limited rural transportation system (10).

Mississippi County is a predominately agricultural community (11); approximately 35% of the county's total population is considered rural (11). The population of Mississippi County is 46,741; of this number 36% are minorities (some race other than white), and a high percentage is low-income (27% below poverty level vs 14% nationally) who either have no health insurance or are underinsured (12-14). Approximately 26% of residents have less than a high school education (vs 16% nationally) (13). Representing the county is Mississippi County Arkansas Economic Opportunity Commission, Inc (MCAEOC), a nonprofit organization committed to enabling low-income residents of Mississippi County to become self-sufficient.

Approximately half of the population in St. Francis County is rural (15). Also a predominately agricultural community (15,16), St. Francis County has a population of 26,783; of this number 54% are minorities, and a high proportion are low-income (32% below poverty level vs 14% nationally) who either have no health insurance or are underinsured (12,14,17). Approximately 26% of residents have less than a high school education (vs 16% nationally) (17). Representing the county is East Arkansas Enterprise Community (EAEC), a nonprofit rural development program that assists communities in St. Francis County through financial and technical support.

Both Mississippi and St. Francis counties have striking racial disparities in colorectal cancer deaths; African Americans in Mississippi (43.7 per 100,000 population per year from 1997 to 2007) and St. Francis counties (37.3 per 100,000) have higher age-adjusted colorectal cancer death rates than do whites in Mississippi (22.1 per 100,000) and St. Francis counties (26.1 per 100,000) (18).

ECL arose from a 9-year partnership starting in 2001 between the University of Arkansas for Medical Sciences (UAMS) and 9 community-based coalitions organized as regional cancer councils representing 10 of Arkansas's 75 counties. Cancer councils, originally funded by the Centers for Disease Control, identify cancer-related problems in their local communities, establish local cancer control priorities, identify and fill gaps in local service and delivery, improve communication with local health care providers, and develop intervention strategies that fit their community's unique needs. UAMS collaborates with the cancer councils through a participatory approach in assessing the assets and needs of the coalition and in developing a research agenda responsive to community interest and priorities.

## Methods

### Building the ECL partnership

In 2006, the partnership received funding from the National Cancer Institute for pilot research projects to strengthen and broaden its networks across the state. The St. Francis and Mississippi cancer councils implemented pilots focused on colorectal cancer, which was the issue of interest identified by both councils. The lead organization in the St. Francis County cancer council was EAEC. The lead organization in the Mississippi County cancer council was MCAEOC. Academic partners met regularly with community partners of awarded cancer councils to implement the pilots. Data from the pilots resulted in the development and funding of ECL.

ECL is a CBPR intervention designed to increase colorectal cancer screening rates via FOBT among adults aged 50 years or older who do not adhere to screening guidelines. The intervention is based on social cognitive and diffusion theories. The objectives of the partnership were to use a CBPR approach to build infrastructure to conduct research, design materials and methods salient to the community, recruit and train lay health advisors and role models, and develop an assessment instrument. The goal is to test the efficacy of ECL in a 5-year randomized controlled trial with 750 participants who do not meet colorectal cancer screening guidelines. The study is approved by the UAMS institutional review board.

Each partner had negotiated subcontracts, which gave the community visible power and equity and set the stage for shared decision making (7). To create a strong sense of ownership, partners named the study Empowering Communities for Life. Community partners say they hope to empower members of the community through education about the benefits of screening to prevent colorectal cancer.

The partnership represents the target community in several ways. Representatives from MCAEOC are Mississippi County natives and consist of 2 African American women and 1 white man. Representatives of EAEC are St. Francis County natives and consist of 2 African American women. University partners include 5 African American women, one of whom is a Mississippi County native, 2 African American men, 3 white men, and 1 Asian woman, for a total of 11 academic partners. Beginning in August 2008, the diverse partnership worked together for 9 months to develop ECL (Figure).

**Figure:**
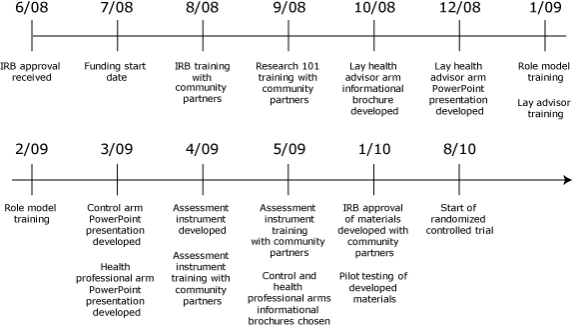
Timetable of major milestones, Empowering Communities for Life program, Mississippi and St. Francis counties, Arkansas. Abbreviation: IRB, institutional review board.

### Building research infrastructure

In initial ECL meetings, the community partners were less vocal than academic partners in discussions of study design and intervention development. When asked, the community partners said that they were unfamiliar with many of the research terms used. To facilitate equitable collaboration, an academic partner used previously developed materials in her work with Lower Mississippi River Delta communities to develop a 4-hour training in basic research for community partners. Academic partners also developed an 8-hour training session in certification to perform research with human subjects to supplement a computerized UAMS training program, which used technical terms unfamiliar to the community partners. All community partners participated in the training.

### Developing ECL materials and methods

Intervention materials were developed for 2 intervention arms and a control arm. The lay health advisor arm consists of a PowerPoint presentation about colorectal cancer delivered by a lay health advisor, a corresponding brochure developed with community partners that reinforces the main points of the presentation, and a community member's (role model's) 3- to 5-minute testimony about his or her experience with colorectal cancer screening. The health professional arm consists of a PowerPoint presentation about colorectal cancer delivered by a health professional and a corresponding brochure from the American Cancer Society. The control arm consists of a presentation about cardiovascular disease delivered by a health professional and a corresponding brochure from the American Heart Association. Recipients of the intervention will be adults in Mississippi and St. Francis counties aged 50 years or older who are not adherent to colorectal cancer screening guidelines.

To facilitate collaborative development of intervention components, academic partners presented initial drafts of PowerPoint presentations and brochures. Community partners reviewed the presentations and talked about the power of storytelling in the community. Academic partners described the Witness Project (19), a successful cancer screening program that uses storytelling, as a potential model for the storytelling component. Thus, the partnership decided to have a community member tell his or her colorectal cancer screening story in the lay health advisor arm to provide a model of screening behavior and to give participants a personal perspective on the screening experience. Community partners spoke of the importance of engaging the audience so that the presentation would be interesting to them; thus, the partnership decided to include checklists in the brochures for the lay health advisor arm for readers to indicate their own risk for colorectal cancer and symptoms of colorectal cancer they may have. The partnership also decided that the latter half of the presentation in the lay health advisor arm should consist of an interactive demonstration on how to use the FOBT.

To refine the intervention, community partners practiced delivering the presentation of the lay health advisor arm to all partners, whereas academic partners delivered the PowerPoint presentations of the health professional and control arms to all partners. The academic partners ensured that community partners delivered the information accurately, whereas community partners ensured that the presentations were delivered in a way that would be interesting to the audience. All partners subsequently made revisions to the intervention and control arms. Revisions included the addition of more discussion questions, graphics, and sound effects to the lay health advisor arm presentation. Aspects of each presentation were also changed to enhance clarity. For example, the partnership decided to use peanut butter in the FOBT interactive demonstration to familiarize participants with stool handling. The PowerPoint slides and brochure were fine-tuned iteratively; several rounds of revisions and presentations increased the clarity and accuracy of the information.

### Selecting and training lay health advisors and role models

The partnership chose employees from EAEC and MCAEOC to serve as lay health advisors because of their 1) recognition in the community as providers of trusted advice and support, 2) experience as lay health advisors on previous cancer council projects, 3) ability to be discreet with participants' information, 4) involvement in the project since its inception, 5) interest in project goals and activities, and 6) available time to devote to the project.

Community partners developed a strategy to recruit role models who would present their personal experience with colorectal cancer screening. Role models had to reside in either Mississippi County or St. Francis County, have received some type of colorectal cancer screening in the past year, and provide informed consent. Community partners targeted people whom others naturally turn to for advice, emotional support, and tangible aid, and who were known in the community as being discreet.

Academic partners developed a 20-hour lay health advisor training (19). The training was led by an academic partner, and initial topics included an overview of the project, the role of the lay health advisors in the project, and the importance of confidentiality. The intervention's presentation components were then reviewed in detail. Each component had corresponding PowerPoint slides, presentation notes, and flash cards with questions and answers. The final part of the training included mock presentations by each lay health advisor at community sites. Community and academic partners critiqued the presenter to improve the presentation. Certification to be a lay health advisor required completion of training and passing the final exam with a score of 80% or higher. To maintain the level of competence achieved through the training, lay health advisors met with one another and with academic partners to practice the presentation.

Role models underwent 5 hours of training, which was developed by academic partners and refined by community partners. The training began with an overview of the project, the intervention presentation, the job of the role model, and the importance of confidentiality. A lay health advisor delivered the intervention presentation, which gave a basic overview of colorectal cancer and the importance of screening. Role models were divided into groups, which were co-facilitated by academic and community partners. Each role model was asked to tell his or her story based on a given outline. Feedback was given to each role model. To maintain the level of competence achieved through the training, role models met with community and academic partners to practice the presentation.

### Preparing the assessment instrument

The assessment instrument was created to assess participants' self-reported medical history and preventive health services, knowledge of screening recommendations, and attitudes regarding preventive behaviors. Academic partners presented a list of demographic, behavioral, and psychosocial factors associated with FOBT use for the partnership to decide which factors to include in the assessment. For each factor chosen, the partnership decided which questions to include by using previous questionnaires (20). Community partners said the survey would need to be engaging for participants to give honest answers. The partnership decided to use an audience response system (OptionPower 3.2, Option Technologies Interactive, Orlando, Florida), which presents assessment questions in PowerPoint that participants can answer using a keypad. Academic partners drafted the assessment and trained lay health advisors to use the audience response system; during a series of meetings at which lay health advisors practiced administering the assessment, community and academic partners made revisions to maximize readability and clarity.

## Outcome

Development of ECL helped strengthen the collaborative relationship between the partners (Table). An outcome of ECL was the recruitment of 11 academic and 5 community partners in a collaborative relationship to develop a CBPR colorectal screening intervention. To develop a stronger research infrastructure within the partnership, trainings were conducted to produce human subjects certification and greater engagement among all 16 partners. Training in research methods resulted in the development of a randomized controlled trial design to test the strategies to promote colorectal screening through ECL, for which the return rate for the FOBT will be the primary outcome measure.

Another outcome is the production of theory-based interactive PowerPoint presentations for all intervention arms of ECL that cover the importance of colorectal cancer and cardiovascular disease screenings, production of a brochure for the lay health advisor intervention, and the incorporation of role models to describe their personal experience with colorectal cancer screening.

To implement ECL, 6 lay health advisors and 23 role models were recruited. All lay health advisors and 22 role models were certified.

The partnership also produced an assessment instrument using an audience response system that evaluates patient experiences in the health care system, colorectal cancer screening behavior and knowledge, cardiovascular disease screening behavior and knowledge, risk factors for cancer and cardiovascular disease, and opinions about cancer and cardiovascular disease prevention.

## Interpretation

Given the distance between community and academic partners (191 miles between UAMS and MCAEOC; 95 miles between UAMS and EAEC), the partnership initially decided to alternate regular meetings with conference calls. However, during the conference calls, community partners were less vocal than academic partners. Given that this was the first large-scale research project both communities had been a part of, there was hesitancy in sharing ideas. Some community partners said that working with the university felt like the "small town" meeting the "big city," which made them uncomfortable contributing to discussions. Thus, community and academic partners decided to meet face to face until university partners developed skills to communicate in a community-friendly way and a level of comfort and familiarity between the partners was achieved, which occurred approximately 6 months into the project. In face-to-face meetings, academic partners discovered that they were able to read body language to see whether their questions were being understood, which allowed for adjustments in how questions were worded. Face-to-face meetings also included visual aids to help community partners understand the research. Dialogue was further facilitated by open discussions of community culture and role-playing activities.

Community partners revealed that the university partners were seen as authority figures who know what is best and should not be questioned. Because of this perception, community partners spoke up only when they felt strongly about project decisions; voicing their opinions at all was the equivalent of shouting them. With this understanding, academic partners learned to listen carefully to community partners and to give great weight to every comment. Academic partners also emphasized the importance of community partners' expertise, whereas community partners learned to view academic partners more realistically.

## Conclusion

To our knowledge, only a few studies have developed a colorectal cancer prevention intervention for an at-risk population using a CBPR approach (8,21,22). ECL is a theory-grounded intervention that builds on community resources to address cancer disparities by increasing colorectal cancer screening in an underserved population. Community-based participatory strategies incorporating sound research methods and health behavior theory have guided the development and implementation of this study. A product of a 9-year partnership, ECL may be a useful model for community-based interventions to increase colorectal cancer screening among rural, underserved groups, and a step toward eliminating disparities in health.

## Figures and Tables

**Table.** Objectives, Methods, and Outcomes of Developing Empowering Communities for Life Program, Mississippi and St. Francis Counties, Arkansas

**Objectives**	**Methods**	**Outcomes**
Develop research infrastructure	Partners receive training in research methods Partners receive training in obtaining institutional review board approval and HIPAA compliance	Greater collaborative engagement within the partnership Certification in conducting research with human subjects and HIPAA compliance Design of the randomized controlled trial to test the intervention's efficacy
Develop intervention materials and methods	Academic partners develop initial drafts to be reviewed by community partners Community and academic partners present intervention materials for revision and refinement	Final intervention materials and methods Strengthened community-academic partnerships
Select and train lay health advisors and role models	Community partners develop initial strategy to recruit lay health advisors for review by academic partners Academic partners develop initial training protocols for lay health advisors to be reviewed by community partners Delivery of training to lay health advisors by all partners	6 lay health advisors recruited 23 role models recruited 6 lay health advisors certified 22 role models certified
Develop the assessment instrument	Academic partners present initial list of evaluation topics; community and academic partners choose final topics Academic partners train community partners in an audience response system Community partners practice delivering assessment instrument, which facilitates revision and refinement of the instrument	Finalized assessment instrument

Abbreviation: HIPAA, Health Insurance Portability and Accountability Act.
